# Typhoidal Orchitis

**Published:** 1909-03-27

**Authors:** 


### Medicine.

### TYPHOIDAL ORCHITIS.

Orchitis in connection with typhoid fever is a complication that is well enough known; nevertheless it is one that may readily be ascribed to another cause, particularly as it most frequently occurs not during the fever but when convalesence is well advanced. The following is a fairly typical case, in which some interesting observations were made by MM. Launois and Loeper.*

### An Illustrative Case.

The patient was a man of forty years of age who presented all the symptoms of a typhoid fever of moderate intensity. The illness ran its usual course, the patient's temperature being restored to normal on the twenty-sixth day of the disease. The rose spots and the Widal's reaction were both well marked. Ehrlich's diazo-reaction was characteristic. The blood count showed typical reduction in the total number of leucocytes and relative diminution in the polymorphonuclear cells. Ehrlich's reaction began to fade on the nineteenth day and it had gone by the twenty-third.

Cure seemed to be complete when on the thirty-eighth day from the beginning of the illness there was a sudden rise of temperature to 103.4° F. The patient stated that during the previous night he had developed acute pain in his left testicle, with a sense of extreme fulness in it. On examination this testicle was found to be swelled to twice the size of the other. It was in a state precisely similar to that produced by gonorrhœal epididymo-orchitis.

There was not a sign of urethral discharge, nor had the patient ever had any. That the orchitis was undoubtedly typhoidal was shown conclusively by bacteriological examination later. The activity of Widal's reaction had simultaneously increased. Fluid obtained by puncture from the tunica vaginalis also clumped cultures of typhoid bacilli with as much readiness as did the blood serum. The leucocytes had resumed characters typical of typhoid fever, the counts being as follows:

| | | |
|---|---|---|
| Total leucocytes per cub. mm. of blood | | 6,500 |
| Differential leucocyte count: | | |
| Mononuclears | ... ... | 52 per cent. |
| Polymorphonuclears | ... | 48 per cent. |
| Eosinophiles | ... ... | absent. |

Ehrlich's reaction had also returned. Pyrexia continued for eleven days, the temperature gradually falling to normal as the testicle, though still swollen, became less painful. On the eighth day of the orchitis the testis was punctured with all aseptic precautions, a small amount of blood being obtained and cultivated. Abundant growths of bacillus typhosus resulted, clumping readily in the patient's diluted serum.

Notwithstanding that the temperature remained normal a portion of the left testicle broke down, suppurated, and discharged pus through a fistulous opening in the scrotum. Some of this pus was cultivated, and it likewise yielded pure cultures of typhoid bacilli. Films of the pus contained many of the bacilli and an abundance of leucocytes, of which the mononuclears were far more abundant than is usually the case in pus due to streptococci or to pneumococci. Cicatrisation resulted in due course, but the patient was not well until the fifty-second day from the beginning of the orchitis, or ninety days from the beginning of the fever.

---

The orchitis in the above case followed the rule in that it occurred during convalescence. Châlon showed this to be true of twenty-two cases out of twenty-nine. It is unusual for it to develop so soon as the sixth day after the fall of temperature to normal. The onset is remarkably sudden. It is an orchitis proper and not an epididymitis only. Suppuration occurs in about a quarter of the cases, the remainder resolving without obvious pus formation. The duration of the orchitis for fifty-two days is exceptional, but it serves to illustrate the fact that as a complication of typhoid fever, it is by no means to be treated lightly.

### PATHOLOGY.

As to the pathology of the condition, it is nowadays regarded as the result of blood infection by the bacilli. Typhoid bacilli during the fever are to be found in the blood in almost every case. It is surprising perhaps that they do not attack the periosteum, the gall-bladder, the lung, the testicle, and so on in more cases than they do. The fact that the hydrocele fluid contained agglutinins is worthy of note, especially as its power of clumping the typhoid bacilli was approximately equal to that of the patient's blood serum. One might have expected that, if agglutinins reached the hydrocele fluid at all, they would do so directly from the bacteria in the testicle, in which case the clumping power of the hydrocele fluid might have been expected to be greater than that of the blood serum at the same time.

It is also noteworthy that the blood-leucocyte count during the orchitis resembled that of typhoid fever itself, and not that of staphylococcal suppuration. During the fever proper there was no leucocytosis, and the polymorphonuclear cells diminished whilst the mononuclears increased. As soon as convalescence set in there was a recovery of the polymorphonuclear cells and a restoration to normal of the relative proportions of these and of the mononuclears. When the orchitis developed there was again no leucocytosis, and the polymorphonuclear cells, instead of showing a relative increase up to 75 per cent. or even 80 per cent. as in ordinary suppurations, exhibited a relative diminution, whilst the mononuclear cells exhibited a relative increase, just as was the case in the original typhoid fever.

Finally, particular attention may well be directed to the varieties of leucocytes in typhoidal pus. The

* *Bull. et Mém. de la Soc. Méd. des Hôp. de Paris.* Series III. Vol. 17.

microscope showed, in films suitably stained, a superabundance of mononuclear leucocytes. In the above case their number was undoubtedly much greater than would be the case in pus of streptococcal or of gonococcal origin. The relative diminution in the number of polymorphonuclear cells in typhoidal pus is, roughly speaking, proportional to their fall in the differential leucocyte blood-count. Similarly in cases of suppurative periostitis or osteomyelitis there is evidence to show that the histological characters of the pus-films varies with the microbe at work; the pus from post-typhoidal periostitis is stated to contain much larger relative numbers of mononuclear cells than does pus from suppurative periostitis of staphylococcal origin. The pus in the latter condition sometimes consists almost solely of polymorphonuclear cells.

The possible value of information thus derived from observation of the leucocytes in stained pus-films is clearly very great.

